# A Review of Patient’s Knowledge and Practice of Diabetic Foot Self-Care

**DOI:** 10.21315/mjms2024.31.1.3

**Published:** 2024-02-20

**Authors:** Eka Kartika Untari, Tri Murti Andayani, Nanang Munif Yasin, Rizka Humardewayanti Asdie

**Affiliations:** 1 Doctoral Graduate Program, Faculty of Pharmacy, Universitas Gadjah Mada, Yogyakarta, Indonesia; 2 Department of Pharmacology and Clinical Pharmacy, Faculty of Pharmacy, Universitas Gadjah Mada, Yogyakarta, Indonesia; 3 Faculty of Pharmacy, Universitas Gadjah Mada, Yogyakarta, Indonesia; 4 Department of Internal Medicine, Faculty of Medicine, Public Health and Nursing, Universitas Gadjah Mada - Dr. Sardjito General Hospital, Yogyakarta, Indonesia; 5 Pharmacy Department, Medical Faculty of Tanjungpura University, Pontianak, Indonesia

**Keywords:** diabetic foot, foot care, knowledge, self-care aspects, ulcer

## Abstract

Since diabetic foot ulcers (DFUs) are common among diabetes patients, it is essential to increase patients’ knowledge and self-care practices to ensure early recognition and management and reduce amputation risk. Therefore, the goal of this review was to identify the range and level of knowledge of people with DFUs and the type of self-care they undertake. A literature review was conducted using the electronic databases PubMed and Google scholar with ‘diabetic foot’, ‘self-care’, ‘practice’ and ‘behaviour’ as searching keywords. The identification and selection process were conducted to sort the eligible papers through the Preferred Reporting Items for Systematic Reviews and Meta-Analyses (PRISMA). The criteria are the original papers describing knowledge and practice in DFU; reporting knowledge and practice in their non-intervention studies; writing in English language; and publishing between years 2016 and 2022. The eligible papers were assessed using the strength of reporting observational studies in epidemiology (STROBE) checklist for appraising their quality. Twenty-two papers of 2,073 titles met the inclusion criteria and included in the review. The lowest and the highest quality score of included papers based on STROBE checklist are 11 and 26, respectively. The included papers showed various levels of knowledge from good to poor, which prominent the highest percentage are 88% (good knowledge) and 84.8% (poor knowledge). The majority of the foot-care activities found in the reviewed papers involved the following steps: washing, drying, applying moisturiser and trimming nails routinely. Those activity should be followed by checking the feet with a mirror for ulcers, looking for ingrown nails, choosing appropriate footwear, not walking barefoot and routinely consulting a healthcare provider. The knowledge levels were found variable and acceptable. Daily foot care, choosing the right footwear, foot activity and regular health checks should all be used to manage diabetes.

## Introduction

World Health Organization (WHO) reported that approximately 422 million individuals with diabetes globally reside in low- and middle-income nations, and diabetes is directly related for 1.5 million deaths, annually. Over the past few decades, there has been a consistent rise in both the incidence and prevalence of diabetes. Diabetes is a significant contributor to renal disease, heart attacks, strokes, blindness and lower extrimities amputation, which is often the results of infected foot ulcers (1). Diabetic patients about 15% to 25% tend to have ulceration on foot at some day in their live (2). Foot problems such ulcers, infections and amputations are among the most significant and burdensome complications of diabetes mellitus. However, these issues can be avoided with a straightforward action including consistent foot care (3). Especially when it comes to foot care, a diabetes care programme especially foot care needs to place a strong focus on transforming the way healthcare is delivered and established, enhancing care education, expanding access to healthcare, and reinforcing preventative measures to lower the morbidity and mortality of this catastrophic condition (4).

Early recognising in foot ulcer risk, immediate adoption of prevention measures, and rapid and intense treatment of foot issues in multidisciplinary foot care facilities are all possible techniques to reduce the consequences of foot complications (5). The patient has a critical role in the prevention of diabetic foot disease, hence foot care knowledge is essential (6). Patients can protect their foot from injury and infection with proper foot care practices, which can also facilitate early detection of foot problems (5). Patients are more likely to take care of their feet including preventing diabetic foot ulcers (DFUs), if they receive proper education and knowledge about their conditions and treatments (7). Knowledge of foot ulcer care is needed by patient to assess their feet on a daily basic care. Those who perceive their feet to be normal may require intensive education and care (8). Additionally, it has been found that patient education is a key component in minimising the occurrence or recurrence of DFUs. This includes disseminating information on fundamental foot care guidelines, such as recommendations for suitable footwear and wound treatment. Also reinforced should be the significance of proper foot hygiene (4). A survey to evaluate the effectiveness of patient education on diabetic foot care showed that it had a good impact on patients’ health attitudes (9–11), as evidenced by a statistically significant improvement in blood pressure and glycaemic control (12), especially knowledge and behaviours on foot self-care (10, 11). However, evidence suggests that individuals’ assessments of their own diabetes-related foot issues are incorrect (13, 14).

Thus, a detailed systematic review on knowledge and practice of DFU self-care is required to help develop an appropriate education programme for foot ulcer patients. The objectives of this review are to determine the spectrum of knowledge and self-care practices of people with DFUs. The results of this review will serve as a valuable reference for health systems to create education programmes to increase knowledge levels and practices of self-care among DFU patients (15). Such educational programmes could help prevent and reduce the severity of DFUs by adopting recommendations from the review to increase patients’ knowledge.

## Method

Searches for papers concerning the self-care of DFUs were undertaken through the PubMed and Google Scholar databases. Medical subject headings (MeSH) of the search terms were searched in English-language publications: ‘diabetic foot’ [MeSH] AND ‘self-care’ [MeSH]. The MeSH terms were used in the PubMed searching database, while the words ‘diabetic foot’ and ‘self-care’ and ‘practice’ and ‘behaviour’ as terms were used in Google Scholar searches.

Papers met the inclusion criteria if they were original articles or full papers published in English from 2016–2021. Articles that reported self-care practices by people with DFUs were included if they described both the frequency of self-care activities and behaviours and knowledge of self-care. Abstract articles, literature review articles, books, letters to the editor, and studies published in proceedings were excluded. In addition, papers were not included if they reported interventions to improve self-care related to DFU. Intervention studies were excluded since the aim of this paper was to explore DFU self-care practices without the influence of interventions. Only the relevant article that describes the subject’s practice and knowledge of DFU self-care was considered to further review the process. Screening papers based on inclusion and or exclusion criteria which were done by authors. The authors screened papers based on the inclusion and exclusion criteria. Paper abstraction was identified by the author according to publication study, paper field, number of subjects, study design, type of practice or behaviour and level of knowledge on DFU’s self-care. The data abstraction was developed by using Microsoft Excel and Zotero as reference manager to assist in collecting selected papers.

Two authors were EKU and TMA, who reviewed independently the papers by title and abstract based on included criteria. A Preferred Reporting Items for Systematic Reviews and Meta-Analyses (PRISMA) diagram was to detail the identification of papers and the selection process. In a literature search, 2,073 papers were identified. A total of 207 duplicated titles were removed whilst 1,866 papers were assessed for eligibility. Titles and abstracts were reviewed against the inclusion criteria, therefore 1,684 references were excluded. There were 1,234 irrelevant papers that did not include information on knowledge, practice or behaviour of foot self-care by diabetes patient in their assessment. These papers were not relevant to review for the following reasons: diabetes and/or diabetic foot was not the sole discussion; there were other diseases that were covered including diabetic foot; one or more aspects of diabetic foot self-care were assessed (for example, feet examination and footwear); the papers studied disease factors, diabetic foot’s comorbidity factors, factors affecting knowledge and practice of self-care, such as psychology factors, perceiving of disease factors, ethnic and characteristic factors; and the papers’ discussed reducing the risk of foot ulcers by developing a prevention programme or other preventive tools.

**Figure 1:**
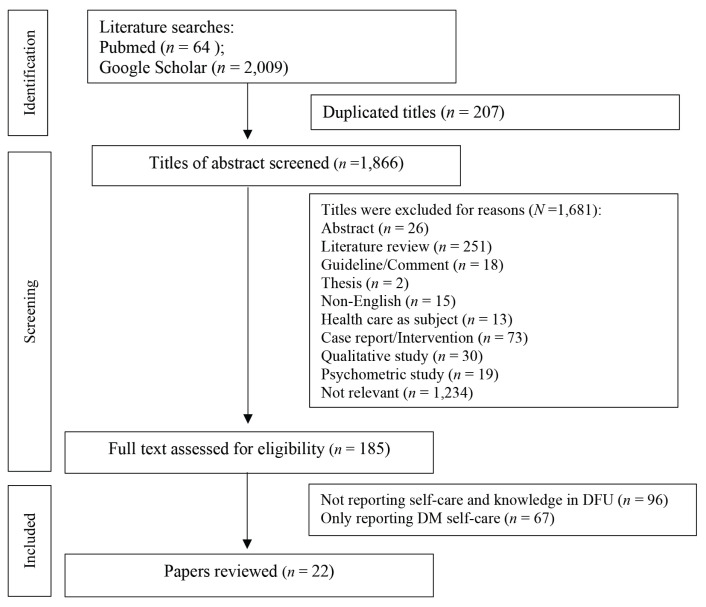
Searching strategy using PRISMA

The full text was retrieved for the remaining 185 articles, resulting in 163 articles being excluded (Figure 1). A total of 22 papers were included in the review process. After the eligible papers were identified through the criteria, the strength of reporting observational studies in epidemiology (STROBE) checklist was used to appraise the quality of the full paper, which was published in 2007. In addition, the authors examined the quality of the papers using the STROBE checklist. The STROBE checklist was used because this review addressed cross-sectional, cohort and case-control studies. The authors used the scores from the checklist, based on STROBE guidelines, to determine the overall quality of the papers. Score of 0 indicates that a particular checklist item is not satisfied, 1 indicates that a specific checklist item is fulfilled and NA indicates that a specific checklist item is not applicable for the specific publication (16).

## Results

### Study Characteristics

Twenty-two papers were eligible for this review after being screened using PRISMA process. The characteristics of included papers were described in Table 1. The distributions of research sites were most in Asia, there was Pakistan as the most frequent country that studying about foot self-care, nonetheless, West Asia is the most country research performed. The number of participants enrolled was ranging from 38–1,030 people. All papers used a cross-sectional design and following the inclusion criteria, many were non-intervention cohort studies. Nine papers using Chi-square test to obtain the association between variables (15, 17–24). Only Sari et al. (33) and Pourkazemi et al. (15) using multivariate regression, meanwhile, correlation test was analysed by Sen et al. (24) and Pourkazemi et al. (15). Other types of analysis used to obtain the differences between groups were the *t*-test, Wilcoxon, Mann-Whitney U and ANOVA tests (15, 16, 25, 26).

Participants who were diagnosed with only type 1 or type 2 diabetes mellitus were enrolled in these included studies, but most papers stated that both type 1 and 2 diabetes mellitus patients participated. Some studies involved participants with a history of ulcer or who currently had ulcer. The papers reported knowledge and practice of DFU self-care which were assessed by varied questionnaires as tools. However, some papers stated the type or name of the tool to measure knowledge and practice was unclear, they measured the knowledge and practice on a Likert scale or the mean number or frequency of times on the reported items. There were common specific questionnaires used to measure knowledge and practice of diabetic foot such as the Foot Care Practice Assessment Questionnaire, Modified Diabetic Foot Care Knowledge (MDFCK) and Modified Diabetic Foot Care Behaviours (MDFCB), the Nottingham Assessment of Functional Foot Care (NAFFC), Diabetes Knowledge Test (DKT) and Diabetes Foot Care Questionnaire (DFQ), and the Diabetic Foot Ulcer Scale-Short Form and the Veterans Affairs-Diabetes Foot Care Survey. Some papers stated the tool to assess both knowledge and practice were developed by the author itself and they have been validated or fulfil the reliability test, in addition to the tool that there are papers did not mention clearly what or how the tools were developed (Table 1). Most of the questionnaires tested the good foot-care practices in the areas of feet washing techniques, an inspection of foot and footwear, skin and nail care, footwear use, and self-foot-care management. Those questionnaires measured the knowledge and practices of patients with and without DFUs. Study of the Magbanua (17), Sari (33) and Sulistyo (31) were using illustrations that accompanied the questions to help participants understand the questions. Most the studies use their own national language in order to simplify the purpose of questions.

### Study Quality

All of the studies were evaluated using the STROBE based on their strength and validity. The quality of included studies was evaluated using a modified version of the STROBE technique (27). Table 1 showed the results of criticised papers ranged from 11 to 26 points (out of 29). Studies of Shamim (28) and Mustafa (29) had the lowest scores, they were 11 and 13 respectively (28, 29). Mustafa (29) did not explain how quantitative variables were handled in the analyses and the limitation of their study. Neither Mustafa et al. nor Shamim (28) met the requirements of the categories for statistical methods, participants, indication of missing data or sensitivity analysis. Based on the STROBE checklist, none of the papers used the flow diagram to describe the non-eligible participants and failed to indicate the number of participants with missing data for each variable of interest. The majority of the papers included in this review also failed to explain how missing data were addressed. The majority of the research studies also admitted that there were some flaws with the STROBE checklist.

### Level of Knowledge on Diabetic Foot Care

All of the reviewed papers reported knowledge of DFU self-care in varying interpretations. Most of the papers in Table 1 categorised knowledge into two to three levels—that is, poor-good, unsatisfactory-satisfactory and low (inadequate)-moderate-high (adequate) knowledge, respectively (17–20, 22, 23, 25, 30–32). Other papers measured knowledge in average or mean scores and in percentage or frequency in each domain. Four of the 10 papers revealed that the number of participants who had poor knowledge was more than 50% (15, 17, 18, 33). Ataseven (34) and Sari (33) revealed insufficient foot care knowledge scores. Thus, the rest of the papers showed that their participants mostly had good knowledge of different foot care aspects. The scope of knowledge was assessed predominantly regarding foot care, while others were concerned about footwear, awareness of ulcer foot risk or diabetes developing into foot ulcers and other supporting information to prevent ulcers.

### Diabetic Foot Care Practice Level

The various level of diabetic foot care by diabetic patients were showed in Table 1. Majority of self-care practices were reported in the frequency of practice. Furthermore, some papers recorded the mean score from the practice domain in the questionnaire, and how many percent of participants met the level of practice, such as poor-good; inappropriate-appropriate, inadequate-adequate or never-always (13, 18, 25, 26, 33–35). Out of 23 papers, 10 papers reported the self-care practice of participants were at a low level of practice for more than 50% of cases (13, 15, 18, 20, 24, 26, 36, 37). Sari (33) found an overall poor level of practice. The rest showed well-founded practices of diabetic foot self-care in favourable numbers, but they had not accomplished an ideal number or more than 90% of practice. Table 3 shows that reported types of practice are similar among papers. Foot care is the primary measured part of self-care practice, other topics were about the self-foot examination and choosing the proper footwear.

## Discussion

### Knowledge and Practice Level

In this review, 22 papers studied various levels of knowledge and type of foot care practice. Knowledge is related to the ability of patient in taking care their ulcers and successful caring can decrease the disease progression become foot amputation (38). Based on some papers, knowledge of foot care also correlates with appropriate practice and reveals a strong correlation between high knowledge and good practice (33, 39, 40).

From the results of the assessment of knowledge and practices of diabetic foot self-care (Table 1), there are still papers with unsatisfying numbers, the numbers of good knowledge and practice were lower than the poor one. The considerations of poor knowledge in some countries may be related to illiteracy which become health education boundaries. Furthermore, diabetics may have been negligent and diminished by their diabetes condition, not paying enough attention to it, which prevents them from attempting to learn about their disease. One that was discovered was that patients were not caring for their feet properly because they assumed that foot care did not have to be conducted every day (33). By enhancing patients’ knowledge and abilities to perform appropriate practices on self-care and self-examination of diabetes, especially foot care, was intended to help patients become aware of their condition and encourage positive behaviour that may allow them to at least reduce the risk of complications.

### Reducing Ulcers Development

From compiling questions on instrument of reviewed papers, the themes about knowledge that can increase the risks of ulcer development (Table 2) emerged as being signs and symptoms of developing an ulcer, maintenance of blood glucose level and type of promoted activity in preventing an ulcer. Patients need to be concerned about the development of gangrene, reduced blood flow, loss of sensation and increased foot temperature followed by redness or foot bleeding as signs and symptoms of foot ulcer development. These conditions are related to diabetes complications caused by neuropathy and ischemic (6). Hyperglycaemic conditions can block the production and activation of endothelial nitric oxide synthase, and the reaction of protein with sugars (Maillard reaction)—which is connected to diabetic complications and ageing—may hasten neuropathy and vascular foot changes (6). Therefore, knowledge of how to maintain blood glucose levels is important. The actions to maintain blood glucose levels include conducting blood glucose monitoring, knowing the hyperglycaemia symptoms, taking anti-diabetes regularly and maintaining a healthy diet and activity. A smoking habit was also quite frequent among diabetic patients in several studies (25, 42), which also affected the reduction of blood flow in foot circulation. Thus, smoking should be avoided because it increases the risk of the development of diabetic foot (19, 26). Moderate walking is unlikely to increase the risk of foot ulcers in people with peripheral neuropathy (42).

### Foot Self-Care

Other aspects of patients’ knowledge in self-care from Table 2 illustrate that the foot self-care consists of foot care, foot inspection, footwear and foot ulcer prevention. Knowledge of self-care was assessed, including general foot care in terms of proper foot washing, checking the temperature of water used for washing, drying the toes and in between the toes, using moisturiser cream and gently filing or removing calluses. These foot self-care aspects were mostly the same as those described in the International Working Group of Diabetic Foot (IWGDF) and the American Diabetes Association guidelines on diabetic foot self-care (5, 43).

Before an ulcer forms, diabetic patients usually experience some changes in their feet, such as hyperkeratosis, dry skin and changes in the shape of their nails and feet (6, 44). Their skin tends to reduce subcutaneous hydration or moisture, although no impairment of subcutaneous barrier function is found. Decreasing dry skin and its elasticity can cause the skin to crack, followed by bacterial invasion and infection. The degree of xerosis or dry skin is related to the duration of diabetes, with desquamation and pruritus occurring in patients (45). Self-care, which starts with washing, drying, hydrating and foot-inspecting daily, is able to reduce the risk factors of foot ulcer occurrence. Therefore, preventive action is important to limit ulcers among diabetic patients through good self-care of the diabetic foot. Sari et al. (33) found that the low score obtained for moisturiser use may be due to the fact that most patients do not know that moisturiser should not be applied between the toes. Additionally, some practices for cleaning, drying and moisturising the foot that are recommended are to use talcum to dry the interspace area completely, and practices that should be avoided include adding an irritant soap, soaking the foot for more than 10 min, to prevent dry skin and using the moisturising cream in interspace but applying it on dry skin immediately after washing. Patients with diabetes need to keep the area between their toes dry using talcum powder and avoid the application of lotion since it is important as a hygienic measure for feet in preventing fungal infection (15, 44). Patients should also use skin moisturisers daily to keep the skin of their feet soft.

Removing the callus should be done by filing it gently. This activity is necessary on a frequent basis (preferably at each doctor visit, as it is crucial for avoiding pressure ulcers) (6). However, patients were found to utilise improper tools to remove calluses and corns. These practices can cause injuries to the feet and lead to infections and other problems.

### Foot Inspections

The knowledge and practices of foot inspections include examining the foot every day and using a mirror to help examine. These practices are intended to find out the foot condition whether any ulcer or not, observe the ingrown nail, any bleeding or redness after removing shoes and socks (footwear), and symptoms of developing an ulcer. Mohamad et al. (35) suggested the proper condition in foot inspection is under good light and during and after showering. Furthermore, Khunkaew et al. (13) recommend to look at the bottom of the feet and check between toes in order to identify any injury and signs of mycosis infection.

### Nail Care

Cutting nails properly and trimming also become an important part of self-care. Cutting nails is recommended to be done at least once a week and trimming nails is allowed by chiropodists (17, 35). Cutting nail is the common activity to cut off the nail, but in diabetic foot self-care trimming nail specifically cutting the nails straight across. The toenails should be trimmed straight across (not rounded) to prevent damage to their toes (43). Thus, patients who are unable to trim their nails should be referred to a podiatrist.

The patient’s knowledge of footwear type is also finds in this review (Table 2). To maintain feet in health condition and keep it from injury, wearing shoes is one of the most crucial act of foot care (46). Using the appropriate footwear is important to protect the foot from injury and to protect the foot from hazardous things, such pins, crushed rocks, broken glass, etc. Therefore, the information about the proper type of footwear, how to select shoes and socks, and how to measure feet to adjust the shoes are essential to complement diabetic foot self-care (34, 36). An appropriate footwear described is not pointy shoes, tight and closed sandals or shoes, comfortable footwear and well-fitting when it is used. Some research found the low knowledge and practice regarding footwear might a result of the knowledge about the use of footwear inadequately and the climate of their country, especially tropical country, so they prefer to use sandals instead of shoes (33).

### Footwear

In Table 3, several papers reported types of footwear were broad, sandal, soft heels, and made of leather and rubber materials (21, 29). Fungi can grow in warm and damp environments, so shoe materials should be permeable to this temperature and humidity (42). The toes should be able to flex freely in shoes. When the patient is standing, they need to be wide enough to prevent strain on the joints and long enough to leave 1 cm between the longest toe and the shoe’s edge (46).

Patients should remain wear socks while wearing shoes to prevent skin irritation and blisters (42). The suitable material of socks for preventing ulcer among diabetics is cotton but nylon is not allowed to use (17, 23, 37). The footwear materials that may rub the feet and causes irritation should be avoid, nevertheless the good shoes are made from flexible and breathable materials. The socks should be changed and washed every day to keep the foot in clean condition and prevent infection (26).

### Performing in Foot Ulcer Prevention

Tables 2 and 3 represent aspects of knowledge and practice in foot ulcer prevention from included studies. These aspects include walking barefoot, manner of sitting and standing, checking the shoes and socks for harmful objects, antiseptics and medication usage, hot bottle or harm objects on feet usage, first aid of fall and injury, choosing proper footwear and visiting the professional for a foot problem. Additionally, patients are advised no to walk without any shoes or sandals in order to prevent the risk of injury.

All papers mentioned that walking barefoot is a harmful activity for diabetes patients. Some papers have suggested using footwear all the time not only outdoor but also when patients are indoors and wearing slippers after washing (20, 24). The wrong position of sitting and standing activities become elements because they reduce foot blood flow and are thus relevant to neuropathy complications. Diabetes patients with or without ulcer and amputation history must not sit with crossed leg and stand for longer time (19, 32, 34). Checking inside of shoes if there were any object or torn lining that could injure the foot is essential to do before wearing footwear, this matter also accomplish for socks. Similarly, the footwear used must be special, it is appropriate and adjusted to the feet (21, 32). If deformation of foot appears, patients have to correct the type and size of their shoes (19). If the shoes were damage or torn, it has to be changed and never being used anymore (26). Other hazardous activity for diabetes patients is put the hot object such as hot water bags or using heater tools to warm their feet. This activity can increase foot temperature and leads inflammation and neuropathy events which end with ulcer formed (47).

A diabetes patient needs to be educated about the management of when they fall or experience an injury so that they can minimise their foot problem become greater (34). The incidence of falls can induce foot deformity, inflammation, and infection. Moreover, diabetes patient who has insensate feet has a greater risk for falls and the disease will worsen (48). In order to prevent foot ulcer complications, Table 2 describes that patients should also have the knowledge of the importance of consulting professional care. Patients will gain the courage to manage their foot-care practices after receiving advice by visiting physician (23). They are also expected to be able to respond to foot problems by consulting professional care (25). Some papers suggest that visiting a professional when patients encounter the diabetes-related problem is important (15, 19, 26, 41). This method should also be applied monthly instead there a foot problem found.

Patients with diabetes can visit the physician to consult routinely about their disease—once a month, every six months, every year, or only during illness (26). Patients could report problems not only if they had an injury or bleeding but also finding the symptoms or signs of the wound on foot including a change in skin crack, colour and temperature. However, using drugstore chemicals, remedies or plasters without prescription on wounds, calluses and corns is a prohibited activity (13) but applying an antiseptic at the first sign of infection is allowed (19, 29).

The findings from this review suggest that mostly DFU patients have undertaken the practices of self-care. This systematic literature review is a thorough examination of foot self-care knowledge and practice, primarily in people with foot ulcers. The review sheds light on an aspect of foot ulcer practice and patient knowledge beyond any intervention that has been carried out in previous research. The research included in this systematic literature review demonstrates satisfactory knowledge and practice. Patients with a higher level of self-care knowledge showed greater adherence to foot care with an emphasis on daily self-care. Diabetic patients who are educated properly could improve their preventive foot care behaviours to decrease the chance of amputations and lower the number of DFU incidents. Based on clinical practice recommendations for diabetes mellitus, patients should receive foot ulcer prevention training in the community and in hospitals. Additionally, amputation was linked to other disease-related factors, knowledge of foot care and sociodemographic features, including the length of the disease, usage of insulin, foot examination, footwear preferences and location in rural or urban areas. These outcomes could be attributed to delayed diagnoses, low healthcare service, and patient-staff communication issues (21).

The current study supports the view that, every time a patient visits, a diabetes educator should provide them with the information they need to help them understand the disease, arrange dietary and lifestyle adjustments, control their blood sugar levels and avoid diabetes complications (15).

The limitations of this review are that it only considered research on foot care knowledge and practice among patients. The systematic review also excluded studies that studied caregivers’ or healthcare providers’ foot care knowledge and practices. However, the reviewed papers demonstrated that the majority of DFU patients have good knowledge and practices of self-care. Additional guidelines required to avoid particular practices should be described to patients so that they are informed, in addition to providing diabetes patients with proper foot care training. It is also necessary to develop a continuous programme to improve the good knowledge and practices that have been established with DFU patients. Daily foot care, choosing footwear, foot activity and consulting with professional health care regularly were all utilised to varying extents to manage diabetes and its complications. Future studies should focus on the impact of knowledge level and practices on quality of life across diverse populations and intervention settings.

## Conclusion

According to the findings of this study, the papers have a range of knowledge categorically, from high to low (high, moderate and low). The highest percentages of high and low knowledge level were 88% and 82.8%, respectively. This investigation also discovered that the prevalence of self-care for diabetic feet is predominantly at a moderate to low level. Along with knowledge, the patients’ practice of DFU’s self-care was apparently good but in some aspects of foot care, they did not fulfil it completely. It can also be concluded that the aspects of the patients’ need for which knowledge is required include information about ulcer development and prevention, glucose level maintenance, foot activity and appropriate daily foot care. Patients should have knowledge regarding foot care, foot inspection, footwear and ulcer prevention activity as well as accomplish good self-care practices.

## Figures and Tables

**Table 1:** Characteristic of reviewed studies

No.	Study	STROBE score	Subject and study design	Site	History and present of ulcer	Diabetes complication	Instrument	Results	
								Knowledge	Practice
1.	Thenmozi and Munya (30)	19	DM Type 1 and 2; *n* = 60; Cross-sectional	Teaching hospital, India	Not mentioned	–	Unclear	Inadequate: 47%; Moderate: 38%; Adequate: 15%	Poor: 43.33%; Good: 41.67%; Best: 15%
2.	Mustafa et al. (29)	11	Not specific; *n* = 90; Cross-sectional	Diabetes centre, Pakistan	Not mentioned	–	Unclear	Good: 88%; Not good: 12%	–
3.	Ataseven and Namoglu (34)	21	Not specific; *n* = 150; Cross-sectional	Private hospital and haemodialysis centre, Turkey	Yes	HD	Foot Care Practice Assessment Questionnaire	–	Score: 57.1 ± 12.1
4.	Mohamad and Lafi (35)	19	Not specific; *n* = 75; Cross-sectional	Public clinic, Iraq	Not mentioned	–	Developed by author	Score: 41.36 ± 5.851	Score: 31.96 ± 5.569
5.	Batista et al. (18)	21	DM Type 2; *n* = 197; Cross-sectional	Primary health care, Brazil	No	–	Diabetes Self-Care Activities Questionnaire (DSQ) and adapted to the Brazilian culture of the Summary of Diabetes Self-Care Activities (SDSCA)	Moderate: 15.2%; Low: 84.8%	–
6.	Sulistyo et al. (31)	17	Not specific; *n* = 81; Cross-sectional	Primary health centre, Indonesia	No	Neuropathy, Perifer arteri disease, Foot deformity	Modified Diabetic Foot Care Knowledge (MDFCK) and Modified Diabetic Foot Care Behaviours (MDFCB)	Poor: 39.5%; Medium: 58%; Good: 2.5%	Poor: 86.4%; Medium: 13.6%
7.	Sari et al. (33)	24	DM Type 2; *n* = 546; Cross-sectional	Primary health centre, Indonesia	Yes	Periferal neuropathy	Foot Care Knowledge (FCK) questionnaire and Modified Diabetic Foot Care Behaviours (MDFCB)	Score: 47.4	Score: 5.33
8.	Khunkaew et al. (13)	17	Not specific; *n* = 41; A cross-sectional study	Diabetes and Foot Clinic, Thailand	Yes	–	The Diabetic Foot Ulcer Scale-Short Form and the VA-Diabetes Foot Care Survey	Unknowledgeable: 65.9%	–
9.	Sutariya and Kharadi (19)	18	Not specific; *n* = 103; A cross-sectional study	Outpatient Surgery Department, India	yes	–	Developed by author	Good: 23%; Satisfactory: 50%; Poor: 27%	Poor: 51%; Good: 33%; Satisfactory: 15%
10.	Qasim et al. (20)	19	Not specific; *n* = 150; Cross-sectional	Outpatient hospital, Pakistan	No	–	The International Working Group on the Diabetic Foot and International Diabetes Federation	Good: 32.7%; Moderate: 51.3%; Poor: 16%	Good: 12.2%; Moderate: 63.3%; Poor: 24.5%
11.	Pourkazemi et al. (15)	23	DM Type 2; *n* = 375; Cross-sectional study	Hospital, Iran	Yes	Not specific mentioned	Standardised questionnaires	Score: 8.63 ± 2.5 Poor: 84.8%	Score: 7.6 ± 2.5 Poor: 49.6%
12.	D’Souza et al. (32)	26	DM Type 2; *n* = 160; Cross-sectional	Public hospital, Oman	Yes	–	Diabetes Knowledge Test (DKT) and Diabetes Foot Care Questionnaire (DFQ)	–	Poor: 18% Good: 82%
13.	Karadağ et al. (21)	21	DM Type 1 and 2; *n* = 1,030; A cross-sectional	Medical Faculty Hospital, Turkey	Yes	Neuropathy	Developed by author	–	Bad: 29.51%; Moderate: 49.61%; Good: 20.87%;
14.	Ahmed et al. (22)	22	DM Type 1 and 2; *n* = 150; Cross-sectional	Diabetes centre, Sudan	Not mentioned	Retinopathy, numbness and tingling, nephropathy	Direct interview by using pre- designed standardised questionnaire	Poor: 20.7%; Moderate: 24%; Good: 46.7%	Poor: 20.7%; Moderate: 36.7%; Good: 42.6%
15.	Sen et al. (24)	24	DM Type 2; *n* = 140; Cross-sectional	Hospital, Vietnam	Not mentioned	–	The Nottingham Assessment of Functional Foot care (NAFF) and Foot Care Knowledge	Knowledgeable: 70%	–
16.	Habbash et al. (25)	24	DM Type 1 and 2; *n* = 400; A cross-sectional	Primary health center, Bahrain	Yes	–	Questionnaire adopted from a previous study (Pollock RD, Unwin NC, Connolly V)	Poor: 8.87%; Desirable: 45.28%; Good: 45.84%	Poor: 41.77%; Desirable: 27.17%; Good: 31.05%
17.	Rabnawaz et al. (41)	18	DM Type 1 and 2; *n* = 380; A cross-sectional	Pakistan	Not mentioned	–	Diabetic Foot Disease (DFD)	–	Good: 41.4%; Poor: 58.6%
18.	Shamim et al. (28)	13	DM Type 1 and 2; *n* = 150; A cross-sectional	Pakistan	Not mentioned	–	Not specific	Score 8 out of 11	–
19.	Abdulghani et al. (23)	24	DM Type 2; *n* = 360; Cross-sectional	Hospital, Saudi Arabia	Yes	Retinopathy, Toe amputation	Developed by author	Poor: 67.9%; Satisfactory: 30%; Good: 2.1%	Poor: 42.9%; Satisfactory: 47.4%; Good: 9.7%
20.	Abo Deif and Abdelaziz (37)	22	DM Type 1 and 2; *n* = 541; Cross-sectional	General hospital, Egypt	No	–	Developed and modified from other authors	Knowledgeable: 75.3%	Good: 33.62%
21.	Magbanua and Lim-Alba (17)	24	DM Type 1 and 2; *n* = 330; Cross-sectional	Tertiery hospital, Phillipines	Yes	–	Knowledge questionnaire developed by Hasnain and colleagues (49); and the Nottingham Assessment of Functional Foot Care (NAFFC)	Good: 82.7%; Satisfactory: 13.3%; Poor: 3.9%	Good: 22.4%; Satisfactory: 71%; Poor: 6.4%
22.	Samia and Tork (26)	19	DM Type 2; *n* = 500; Cross-sectional	Diabetes centre and hospital, Saudi Arabia	No	Heart disease, hypertension, dyslipidaemia, thyroid disease, anaemia, kidney disease	Unclear (only mentioned KAP questionnaire, 2017)	Unsatisfactory: 64%	Inadequate: 56.6%

**Table 2:** Knowledge on diabetic foot self-care

Aspects	Item assessed	Reference
Related developing of ulcer	Developed of gangrene	(26, 28, 29, 35)
	Reduced blood flow	(26, 28, 35, 41)
	Loss of sensation	(26, 28, 41)
	Smoking habit	(19, 25, 26, 28, 37, 41)
	complication disease	(25, 26, 28, 31, 32, 34)
	Foot temperature, redness or bleeding	(37)
Maintenance blood glucose	Blood glucose monitoring	(32)
	Hyperglycaemia symptoms	(34, 35)
	Taking medicine	(19, 32, 34, 37)
	Diet	(32, 34)
Activity	Exercise	(34)
	Foot exercise	(34)
Foot care	Wash feet everyday	(13,19–21, 25, 29, 31, 32, 37, 41)
	Check on water temperature for washing feet (using the lukewarm water)	(13, 17, 19, 20, 25, 29, 34, 35, 37)
	Dry well toes and in between	(15, 17, 19, 20, 29, 32)
	Use moisturising cream/lotion	(13, 19–21, 29, 33, 34, 41)
	Gently filling calluses or removing callus	(13, 19, 20)
Foot inspection	Examine feet every day	(13, 19–21, 25, 29, 32–34, 37, 41)
	Using special mirror	(13)
Nail care	Cutting nail	(19, 20, 26, 29)
	Trimming nail	(15, 21, 29, 32)
Footwear	Type of shoes	(21, 29)
	Type of socks	(29)
	Washing and changing socks	(15, 20, 21)
Foot ulcer prevention	Never walk bare foot or always using footwear	(15, 19–21, 29, 31)
	Never sit crossing leg and standing longer time	(19)
	Check the shoes and socks for foreign objects or torn lining	(19–21, 29, 37)
	Use antiseptics or medication	(19, 20, 29)
	Avoiding hot bottle/objects on feet	(29)
	First aid of fall and injury	(33, 34)
	Choosing the right footwear	(19, 20, 33, 34,
	Visit the professionals for foot problem	(15, 19, 20, 25, 37, 41)

**Table 3:** Practice of diabetic foot self-care

Aspects	Item assessed	Reference
Foot care	Wash feet every day	(17, 19–21, 23–26, 29, 31, 32, 35, 37, 41)
	Check on water temperature for washing feet (using the lukewarm water)	(13, 19, 20, 23, 26, 29, 32, 34, 35, 37)
	Dry well toes and in between	(13, 15, 17–20, 23, 24, 26, 29, 32, 35)
	Use moisturising cream/lotion	(13, 18, 23, 24, 26, 29, 33–35, 41)
	Gently filling calluses or removing callus	(13, 19, 20)
Foot inspection	Examine feet every day	(13, 15, 17–21, 23–25, 28, 29, 32–35, 37, 41)
	Using special mirror	(13)
Nail care	Cutting nail	(13, 17, 19–21, 23, 26, 28, 29, 31, 35, 37)
	Trimming nail	(13, 15, 25, 29, 32, 35)
Footwear	Type of shoes	(17, 18, 24, 29, 33)
	Type of socks	(17, 24, 29)
	Washing and changing socks	(20, 21, 24)
	Wear socks	(13, 18, 23, 26, 32, 35, 37)
Foot ulcer prevention	Never walk bare foot or always using footwear	(13, 17–21, 23, 29, 31, 32, 34, 35, 37, 41)
	Never sit crossing leg and standing longer time	(19, 32, 34)
	Check the shoes and socks for foreign objects or torn lining	(13, 18–21, 29, 35, 37)
	Use antiseptics or medication	(19, 20, 24, 29, 32)
	Avoiding hot bottle/objects on feet	(17, 24, 26, 29, 32)
	First aid of fall and injury	(26, 33, 34)
	Choosing the right footwear	(19–21, 25, 26, 32, 34, 35, 37, 41)
	Visit doctor when the problem was met	(19, 15, 26, 41)
	Taking anti-DM regularly	(15, 20)
	Avoid smoking	(32)
